# P2X7 receptor contributes to hepatic inflammation and collagen deposition in methionine- and choline-deficient diet-fed mice

**DOI:** 10.3389/fphar.2026.1909005

**Published:** 2026-10-07

**Authors:** Sthefani Rodrigues Batista da Silva, Fabiana Cristina-Rodrigues, Mariana M. Chaves, Isabele Pinheiro Cruz, Lorraine de Oliveira da Cruz, Maria Fernanda Carvalho de Araujo, Soraia das Neves Vieira Cavalcanti, Georgia Correa Atella, Christina Maeda Takiya, Luiz Eduardo Baggio Savio, Robson Coutinho-Silva

**Affiliations:** 1 Laboratory of Immunophysiology, Institute of Biophysics Carlos Chagas Filho, Federal University of Rio de Janeiro, Rio de Janeiro, Brazil; 2 Center for Technological Development in Health, Oswaldo Cruz Foundation, Rio de Janeiro, Brazil; 3 Laboratory of Neuroimmunology, Institute of Biophysics Carlos Chagas Filho, Federal University of Rio de Janeiro, Rio de Janeiro, Brazil; 4 Laboratory of Biochemistry of Lipids and Lipoproteins, Leopoldo de Meis Institute of Medical Biochemistry, Federal University of Rio de Janeiro, Rio de Janeiro, Brazil; 5 Laboratory of Immunopathology, Institute of Biophysics Carlos Chagas Filho, Federal University of Rio de Janeiro, Rio de Janeiro, Brazil

**Keywords:** chronic liver disease, inflammasome, methionine- and choline-deficient diet, metabolic dysfunction-associated steatohepatitis, P2X7 receptor

## Abstract

**Introduction:**

Metabolic-Dysfunction-Associated Steatohepatitis (MASH) is characterized by hepatic steatosis, inflammation, and progressive liver injury, with growing evidence implicating purinergic signaling and inflammasome activation in disease progression. Here, we investigated the role of the P2X7 receptor and caspase-1/11 signaling in methionine- and choline-deficient (MCD) diet-induced liver injury in mice.

**Methods:**

Mice were fed an MCD diet to induce liver injury, and hepatic steatosis, inflammation, and tissue injury were assessed by histopathological analysis. Hepatic triacylglycerol and cholesterol accumulation, *Il1b* mRNA expression, and IL-1β protein levels were evaluated. The hepatic expression profile of purinergic receptors, including P2Y_1_, P2Y_4_, P2Y_6_, P2Y_12_, and P2Y_14_, was also investigated in wild-type and P2X7- and/or caspase-1/11-deficient mice.

**Results:**

MCD diet feeding induced significant weight loss and marked histopathological alterations, including hepatic steatosis, inflammatory infiltrates, and hepatocellular injury, accompanied by increased hepatic triacylglycerol and cholesterol accumulation. Hepatic injury was partially dependent on caspase-1/11 signaling, whereas hepatic inflammation was partially dependent on the P2X7 receptor. Although *Il1b* mRNA expression increased following MCD diet feeding, this increase was significant only in wild-type mice, whereas increased IL-1β protein levels were observed only in wild-type animals, suggesting that P2X7 receptor signaling and caspase-1/11 may contribute to IL-1β maturation during MCD diet-induced liver injury. In addition, MCD diet feeding promoted marked remodeling of the hepatic purinergic profile, characterized by downregulation of P2Y_1_ and P2Y_4_ receptors and genotype-dependent regulation of P2Y_12_, whereas P2Y_6_ and P2Y_14_ expression remained largely unchanged.

**Discussion:**

Together, these findings demonstrate that the P2X7 receptor and caspase-1/11 play a role in amplifying inflammatory responses and reshaping the hepatic purinergic microenvironment during steatohepatitis progression, highlighting the P2X7 receptor as a potential therapeutic target in chronic liver disease.

## 1 Introduction

Chronic liver disease (CLD) is a growing global public health concern, with Metabolic Dysfunction-Associated Liver Disease (MASLD) being its most prevalent form (Cheemerla and Balakrishnan, 2021). MASLD affects approximately 25% of the adult population worldwide and may progress from simple, asymptomatic hepatic steatosis to irreversible end-stage liver conditions, such as cirrhosis (Friedman et al., 2018; Rinella, 2015). Within this spectrum, metabolic-dysfunction-associated steatohepatitis (MASH) represents a critical transitional stage, characterized by hepatic fat accumulation, inflammation, hepatocellular injury, and varying degrees of fibrosis (Diehl and Day, 2017). If left untreated, MASH may lead to severe liver damage and increase the risk of hepatocellular carcinoma. Given its progressive and potentially life-threatening nature, early therapeutic intervention is crucial. Effective treatment strategies must target the key pathological features of MASH, including reduction of steatosis, suppression of inflammation, and prevention or reversal of fibrosis (Diehl and Day, 2017).

The liver plays a central role in maintaining metabolic homeostasis through processes such as glucose regulation, lipid metabolism, and detoxification (Liu et al., 2017). During liver injury, hepatocytes release damage-associated molecular patterns (DAMPs), such as adenosine triphosphate (ATP), which activate immune responses and contribute to inflammation through activation of purinergic receptors, highlighting the liver’s role in both metabolism and immune signaling (Ulrich et al., 2025; Zhou et al., 2016).

The purinergic signaling pathway involves various purine and pyrimidine nucleotides, which act through two main receptor families: P1 receptors, which are adenosine-selective, and P2 receptors, which preferentially respond to ATP and ADP, although they can also recognize other ATP-related nucleotides (Bours et al., 2011; Ralevic and Burnstock, 1998). These receptors are expressed on the surface of nearly all cell types, including hepatic cells (Ulrich et al., 2025). Among the P2 receptors, the P2X subfamily consists of ionotropic receptors, with P2X4 and P2X7 being the most abundantly expressed in the liver (Fausther et al., 2012). Their distribution varies by cell type: hepatocytes, for instance, express nearly all P2X subtypes except P2X6 (Fausther et al., 2012; Gonzales et al., 2007). Notably, the P2X7 receptor has been implicated in the pathogenesis of several liver diseases due to its role in promoting oxidative stress, inflammation, and fibrogenesis (Vaughn et al., 2012). Experimental evidence shows that pharmacological inhibition of P2X7 receptor can mitigate liver injury in septic models by downregulating inflammatory signaling pathways, reducing cytokine production, preventing sinusoidal cell apoptosis and liver fibrosis (Savio et al., 2017; Huang et al., 2014; Baeza-Raja et al., 2020). Moreover, previous studies have demonstrated the involvement of the P2X7 receptor in the pathogenesis of MASH (Blasetti-Fantauzzi et al., 2017; Das et al., 2013; Dixon et al., 2012; Iracheta-Vellve et al., 2015). However, the downstream mechanisms triggered by this receptor remain not fully understood.

Activation of the P2X7 receptor promotes the maturation and release of interleukin-1β (IL-1β), a key pro-inflammatory cytokine generated through the activation of the NOD-like receptor family pyrin domain-containing 3 (NLRP3) inflammasome complex. This multiprotein complex consists of the sensor molecule NLRP3, the adaptor protein ASC (apoptosis-associated speck-like protein containing a CARD), and the effector enzyme pro-caspase-1, which is activated via proximity-induced autocleavage following inflammasome assembly. Additionally, the NLRP3 inflammasome can be activated by Caspase-11 (Rathinam et al., 2012). The NLRP3 inflammasome has been identified as a crucial mediator of cell death in the inflammatory response associated with steatotic liver disease (Kaufmann et al., 2022; Yu et al., 2022). Given this context, the present study investigates the contribution of P2X7 receptor signaling to hepatic damage and inflammation in mice subjected to a methionine- and choline-deficient diet (MCD), a well-established model of MASH.

## 2 Materials and methods

### 2.1 Animals and ethical statement

Male C57BL/6 wild-type (WT), P2X7^−/−^, and Casp1/11^−/−^ mice aged 8–12 weeks were used in this study. Animals were housed under controlled temperature (22 °C ± 2 °C) and humidity (55% ± 10%) conditions, with a 12 h light/dark cycle and *ad libitum* access to food and water throughout the experimental period. All experimental procedures were approved by the Ethics Committee on the Use of Animals of the Federal University of Rio de Janeiro (CEUA protocol no. 070/22) and conducted in accordance with the guidelines of the National Council for the Control of Animal Experimentation (CONCEA, Brazil). Mice were euthanized by gradual-fill carbon dioxide (CO_2_) inhalation in a dedicated chamber. CO_2_ was introduced at a flow rate corresponding to approximately 20% of the chamber volume per minute, in accordance with CONCEA recommendations for rodent euthanasia. The gas flow was maintained for at least 1 min after clinical death was observed.

### 2.2 Induction of metabolic-dysfunction-associated steatohepatitis (MASH)

Mice were randomly assigned into two experimental groups: (1) control (CTRL), receiving standard rodent chow, and (2) MCD, receiving a methionine- and choline-deficient (MCD) diet. The MCD diet was used to induce MASH through impairment of hepatic lipid export, promoting steatosis and liver inflammation (Marcolin et al., 2011). Animals were maintained on the experimental diets for 6 weeks, with diets replaced twice weekly. Body weight was monitored throughout the experimental period. At the end of the sixth week, animals were euthanized under deep anesthesia, non-perfused, and blood and liver samples were collected for subsequent analyses.

### 2.3 Body and liver weight measurements

Body weight was recorded twice weekly throughout the experimental period. At euthanasia, livers were excised, washed in phosphate-buffered saline (PBS), dried on filter paper, and immediately weighed. Liver weight was expressed in grams.

### 2.4 Biochemical analysis of plasma aminotransferases

Blood samples were collected by cardiac puncture into heparinized tubes and centrifuged at 1,650 × *g* for 15 min at 4 °C to obtain plasma. Alanine aminotransferase (ALT) and aspartate aminotransferase (AST) activities were quantified using commercial kits (Bioclin, catalog numbers K049-6 and K048-6, respectively) according to the manufacturer’s instructions. Absorbance was measured at 340 nm using a microplate reader, and enzyme activities were expressed as U/L.

### 2.5 Histological analysis and liver inflammatory and damage scoring

For microscopic examination, tissues were fixed in 10% buffered formaldehyde and embedded in paraffin. Sections at 4 μm were obtained, stained with hematoxylin-eosin (HE), and with a modified Picro-Sirius red staining (Dolber and Spach, 1987) for the collagen network. HE stained sections were used for grading hepatocyte damage and liver inflammation.

A histological grading score adapted from Liang et al. (2014) and was applied to 15 high-quality images (2048 × 1536-pixel buffer)/animal obtained with a light microscope (Nikon E800, Japan) assisted by a digital camera (Evolution VF, Media Cybernetics, United States) and transmitted to a color LCD monitor in TIFF format. The interface capture software used was Q-Capture 2.95.0, version 2.0.5 (Silicon Graphics Inc.,United States). The histological score considered the following parameters: (1) macrovesicular steatosis and microvesicular steatosis were both separately scored, and the severity was graded based on the percentage of the total area affected: 0 (0%–5%); 1 (5%–33%); 2 (34%–66%); and 3 (>66%). The difference between macrovesicular and microvesicular steatosis was defined by whether the vacuoles displaced the nucleus to the side (macrovesicular) or not (microvesicular). Similarly, (2) the level of hepatocellular hypertrophy, defined as cellular enlargement more than 1.5 times the normal hepatocyte size, was scored, based on the percentage of the total area affected, into the following categories: 0 (<5%), 1 (5%–33%), 2 (34%–66%) and 3 (0.66%), (3) presence of polyploid cells, defined as cells with enlarged nuclei (more than 3 times the normal hepatocyte nucleus size), scored as 0 (absence); 1 (present), and (4) presence of binucleated cells, also scored as 0 (absence) or 1 (present). The sum (Σ) of individual parameters represented the liver damage index.

Liver inflammation considered (1) lobular inflammation defined as the increase in number of inflammatory cells in the sinusoidal area (mixed inflammatory cell infiltrate, consisting of lymphocytes, neutrophils, eosinophils, and Kupffer cells), and scored as 0 (up to 5 cells); 1 (>5 cells), (2) presence of microgranulomas (aggregates of macrophages/Kupffer cells), scored as 0 (not present) or 1 (present), and (3) presence of lipogranulomas (consisting of fat droplets, inflammatory cells, and collagen), also scored as 0 (absent) or 1 (present). The sum (Σ) of individual parameters represented the liver inflammatory index.

### 2.6 Hepatic lipid analysis

Liver tissue samples (30–60 mg) were homogenized in lysis buffer containing 50 mM Tris-HCl (pH 8.4), 1% Triton X-100, 250 mM NaCl, and 5 mM EDTA. Hepatic triacylglycerol and total cholesterol concentrations were quantified using enzymatic colorimetric commercial kits (Doles®, Goiânia, Brazil) according to the manufacturer’s instructions.

For lipid extraction, liver homogenates were subjected to a modified Bligh and Dyer protocol using chloroform:methanol:water (2:1:0.8, v/v/v). Samples were centrifuged at 1,650 × *g* for 20 min at 4 °C, and the organic phases were collected and combined. After phase separation, the organic fraction containing lipids was recovered and dried under nitrogen (*N*_*2*_) flow.

Thin-layer chromatography (TLC) was performed using silica gel 60 plates (Merck, Darmstadt, Germany). Lipids were separated using hexane:diethyl ether:acetic acid (60:40:1, v/v/v) as the mobile phase. Lipid bands were visualized using a charring solution containing 3% CuSO_4_ in 8% H_3_PO_4_ followed by heating at 150 °C for 8 min. Densitometric quantification of lipid bands was performed using ImageMaster TotalLab software.

### 2.7 RNA extraction and gene expression analysis by RT-qPCR

Total RNA was extracted from liver tissue using TRIzol® reagent (Thermo Fisher Scientific, NJ, United States) according to the manufacturer’s instructions. RNA concentration and purity were determined using a NanoDrop Lite spectrophotometer (Thermo Scientific, NJ, United States). Complementary DNA (cDNA) was synthesized from 1 µg of total RNA using the High-Capacity cDNA Reverse Transcription Kit (Thermo Fisher Scientific, NJ, United States).

Quantitative real-time PCR (RT-qPCR) reactions were performed using SYBR® Green qPCR Master Mix (Promega, Madison, WI, United States) in a Mastercycler® Gradient thermocycler (Eppendorf, Hamburg, Germany). Each reaction contained 2 µL of diluted cDNA (1:10) and 300 nM of each primer in a final volume of 10 µL. Thermal cycling conditions consisted of an initial denaturation step at 95 °C for 10 min, followed by 40 cycles of 95 °C for 15 s and 60 °C for 1 min. Melting curve analysis was performed to confirm amplification specificity.

Relative gene expression was calculated using the comparative Ct method (2^−ΔΔCt^), with β-actin (*Actb*) used as the endogenous reference gene. Relative expression values are presented as 2^−ΔΔCt^, whereas statistical analyses were performed using the corresponding ΔCt values. Primer sequences used in this study are listed in Table 1.

**TABLE 1:** Primer sequences used for RT-qPCR analysis.

Gene	Forward primer (5′–3′)	Reverse primer (5′–3′)	Accession number
*Il1b*	TTC​AGG​CAG​GCA​GTA​TCA​CTC	CCA​CGG​GAA​AGA​CAC​AGG​TAG	NM_008361.4
*Tnf*	GGT​CCC​CAA​AGG​GAT​GAG​AAG​TTC	CCA​CTT​GGT​GGT​TTG​CTA​CGA​CG	NM_013693.3
*Tgfb1*	GAG​CCC​GAA​GCG​GAC​TAC​TA	TGG​TTT​TCT​CAT​AGA​TGG​CGT​TG	NM_011577.2
*P2ry1*	GAC​TGA​CTG​GAT​CTT​CGG​GGA	CCA​CCA​CAA​TGA​GCC​ACA​CC	NM_001282016.1
*P2ry4*	ACT​GGC​TTC​TGC​AAG​TTC​GT	AGG​CAG​CCA​GCT​ACT​ACC​AA	XM_006528178.2
*P2ry6*	TGC​TGC​TTG​GGT​AGT​GTG​TGG	GTA​AGG​CTA​TGA​AGG​GCA​GC	XM_011241739.4
*P2ry12*	CAT​TGC​TGT​ACA​CCG​TCC​TG	ACC​TTG​GCA​CAC​CAA​GGT​TC	NM_001357008.1
*P2ry14*	TTT​TGT​CGT​CTG​CTT​TGT​GC	GCA​GCC​GAG​AGT​AGC​AGA​GT	NM_001287120.1
*Entpd1 (CD39)*	AGC​TGC​CCC​TTA​TGG​AAG​AT	TCA​GTC​CCA​CAG​CAA​TCA​AA	NM_009848.4
*Nt5e (CD73)*	CAG​GAA​ATC​CAC​CTT​CCA​AA	AAC​CTT​CAG​GTA​GCC​CAG​GT	NM_011851.4
*Actb*	TAT​GCC​AAC​ACA​GTG​CTG​TCT​GG	TAC​TCC​TGC​TTG​CTG​ATC​CAC​AT	NM_007393.5

### 2.8 Cytokine quantification by ELISA

Liver samples were homogenized in RIPA buffer supplemented with protease inhibitors and centrifuged at 13,000 rpm for 30 min at 4 °C. Supernatants were used for the quantification of IL-1β (#DY201-05) using DuoSet ELISA kits (R&D Systems, Minneapolis, MN, United States), according to the manufacturer’s instructions.

Absorbance was measured at 450 nm using a microplate spectrophotometer. Cytokine concentrations were determined from standard curves and normalized to total protein concentration measured by the Coomassie Blue method. Results were expressed as pg of cytokine per mg of protein.

### 2.9 Statistical analysis

Data are presented as mean ± SEM. Data normality was evaluated using the Shapiro–Wilk test. Comparisons between two groups were performed using the unpaired Student’s t*-*test or Mann–Whitney test, according to data distribution. Multiple-group comparisons were analyzed using two-way ANOVA followed by Tukey’s *post hoc* test. RT-qPCR statistical analyses were performed using ΔCt values. Body-weight data were analyzed using a mixed-effects model for repeated measurements. Statistical significance was defined as *p* < 0.05. All statistical analyses were performed using GraphPad Prism version 8.4.2 (GraphPad Software, San Diego, CA, United States).

## 3 Results

### 3.1 P2X7 receptor and caspase-1/11 contribute to MCD diet-induced liver injury

The MCD diet is a widely used model for inducing MASH, although it does not reproduce typical metabolic features such as weight gain (Rinella, 2015; Friedman et al., 2018). Consistent with previous reports, our data showed that MCD diet induced significant weight loss in mice (Figures 1A,B), which became evident as early as the first week of treatment. Notably, P2X7 receptor-deficient (P2X7^−/−^) and caspase-1/11-deficient (casp-1/11^−/−^) mice fed the MCD diet were not protected from weight loss and displayed a profile similar to that of wild-type (WT) animals (Figures 1A,B).

**FIGURE 1:**
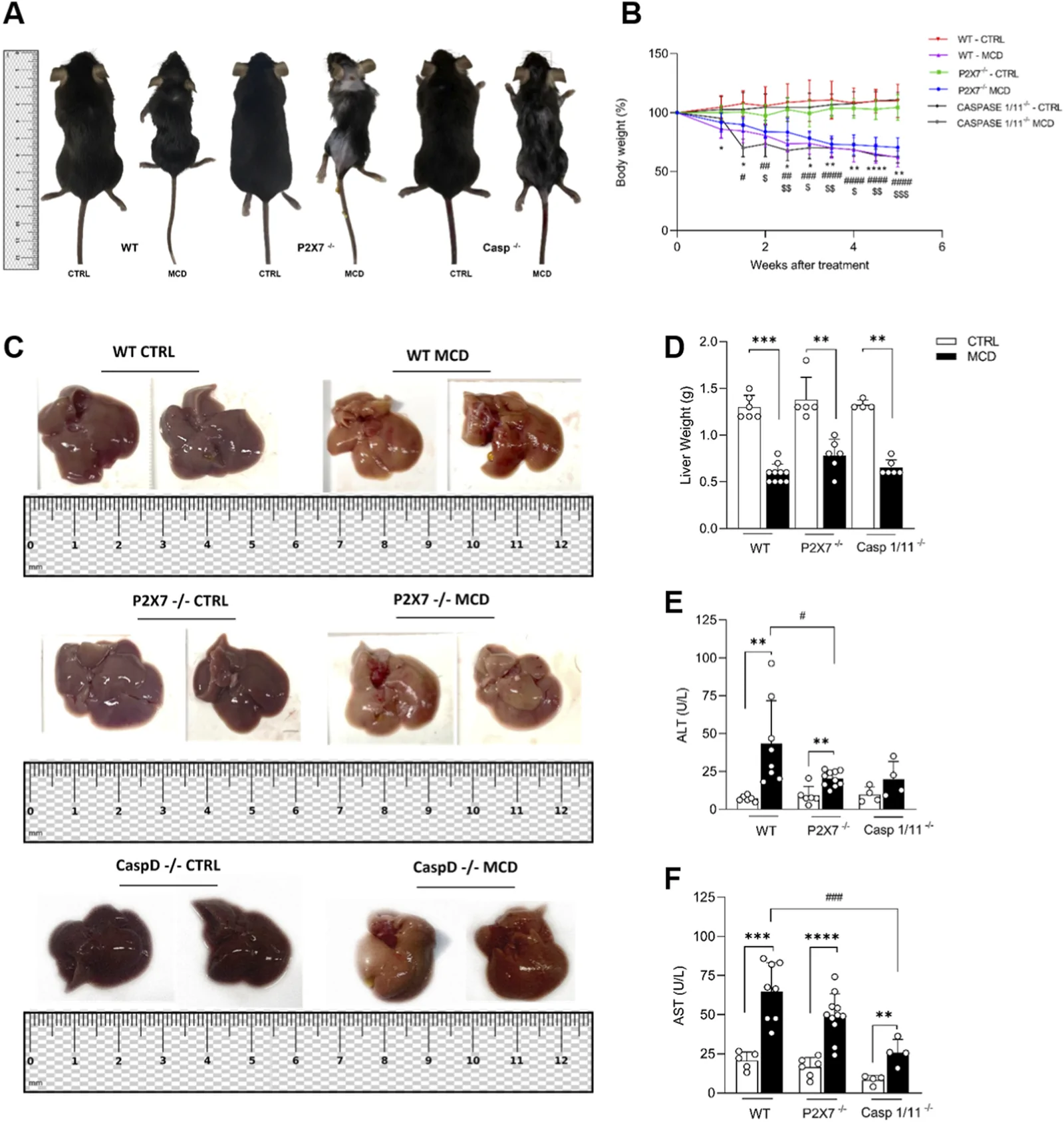
MCD diet induces systemic metabolic alterations and hepatic injury in WT, P2X7^−/−^, and Casp1/11^−/−^ mice. Representative images of mice after 6 weeks of methionine- and choline-deficient (MCD) diet feeding showed a marked reduction in body size in MCD-fed animals compared with their respective CTRL groups **(A)**. Body weight progression demonstrated progressive weight loss throughout the experimental period in all MCD-fed groups, with P2X7^−/−^ and Casp1/11^−/−^ mice exhibiting a pattern and magnitude of weight loss comparable to those observed in MCD-fed WT mice **(B)**. Representative macroscopic liver images revealed paler coloration and reduced liver size in MCD-fed mice **(C)**. Quantitative analysis showed reduced liver weight after MCD diet feeding across all genotypes **(D)**. Serum ALT levels were significantly increased in WT mice following MCD diet administration, whereas no significant increase was detected in P2X7^−/−^ or Casp1/11^−/−^ mice **(E)**. Serum AST levels were significantly increased in WT and P2X7^−/−^ mice, whereas no significant increase was detected in Casp1/11^−/−^ mice following MCD diet feeding **(F)**.

All livers were photographed macroscopically and weighed. A clear difference in liver coloration was observed in all animals fed the MCD diet, whose livers exhibited a more yellowish appearance, a feature associated with hepatic lipid accumulation and injury (Figure 1C). In addition, liver weight was reduced in animals receiving the MCD diet, regardless of the experimental group (Figure 1D). These findings indicate that the effect of MCD feeding on liver weight was similar among genotypes.

Liver injury is commonly associated with increased serum levels of aspartate aminotransferase (AST) and alanine aminotransferase (ALT), enzymes involved in hepatic protein metabolism (Iluz-Freundlich et al., 2020). Analysis of serum enzyme levels revealed a significant increase in ALT in WT mice fed the MCD diet compared with their respective control group, whereas no significant increase was detected in P2X7^−/−^ or caspase-1/11^−/−^ mice (Figure 1E). The ALT response to MCD feeding differed among genotypes, supporting genotype-dependent modulation of this response. WT and P2X7^−/−^ mice fed the MCD diet also showed increased AST levels compared with animals fed the control diet, whereas no significant increase was detected in caspase-1/11^−/−^ mice (Figure 1F). However, the overall AST response to MCD feeding did not significantly differ among genotypes.

Collectively, these results demonstrate that MCD diet induces systemic and hepatic alterations across genotypes, while genotype-dependent modulation was observed for specific markers of liver injury.

Experimental groups included WT CTRL, WT MCD, P2X7^−/−^ CTRL, P2X7^−/−^ MCD, Casp1/11^−/−^ CTRL, and Casp1/11^−/−^ MCD mice. Body weight data were analyzed using a mixed-effects model for repeated measurements, followed by multiple comparisons. Liver weight, ALT, and AST data were analyzed using two-way ANOVA followed by Tukey’s multiple comparisons test. Data are presented as mean ± SEM. Statistical significance is indicated as *P* < 0.05, P < 0.01, *P* < 0.001, and P < 0.0001; ns, not significant. Each data point represents one animal; sample sizes ranged from 4 to 11 animals per group.

### 3.2 P2X7 receptor and caspase-1/11 contribute to hepatic inflammation during MCD diet-induced chronic liver disease

It is already widely known that MASH causes inflammation and hepatocellular injury (Diehl and Day, 2017). Our results demonstrate that WT and P2X7^−/−^ receptor and caspase-1/11^−/−^ mice fed with a control diet had a preserved liver architecture, as expected (Figures 2A,C,E). On the other hand, the MCD diet induces lipid accumulation (macrovesicular and microvesicular steatosis) in hepatocytes and lobular and portal inflammation in the liver of WT and P2X7^−/−^ and Caspase-1/1^−/−^ mice (Figures 2B,D,F).

**FIGURE 2:**
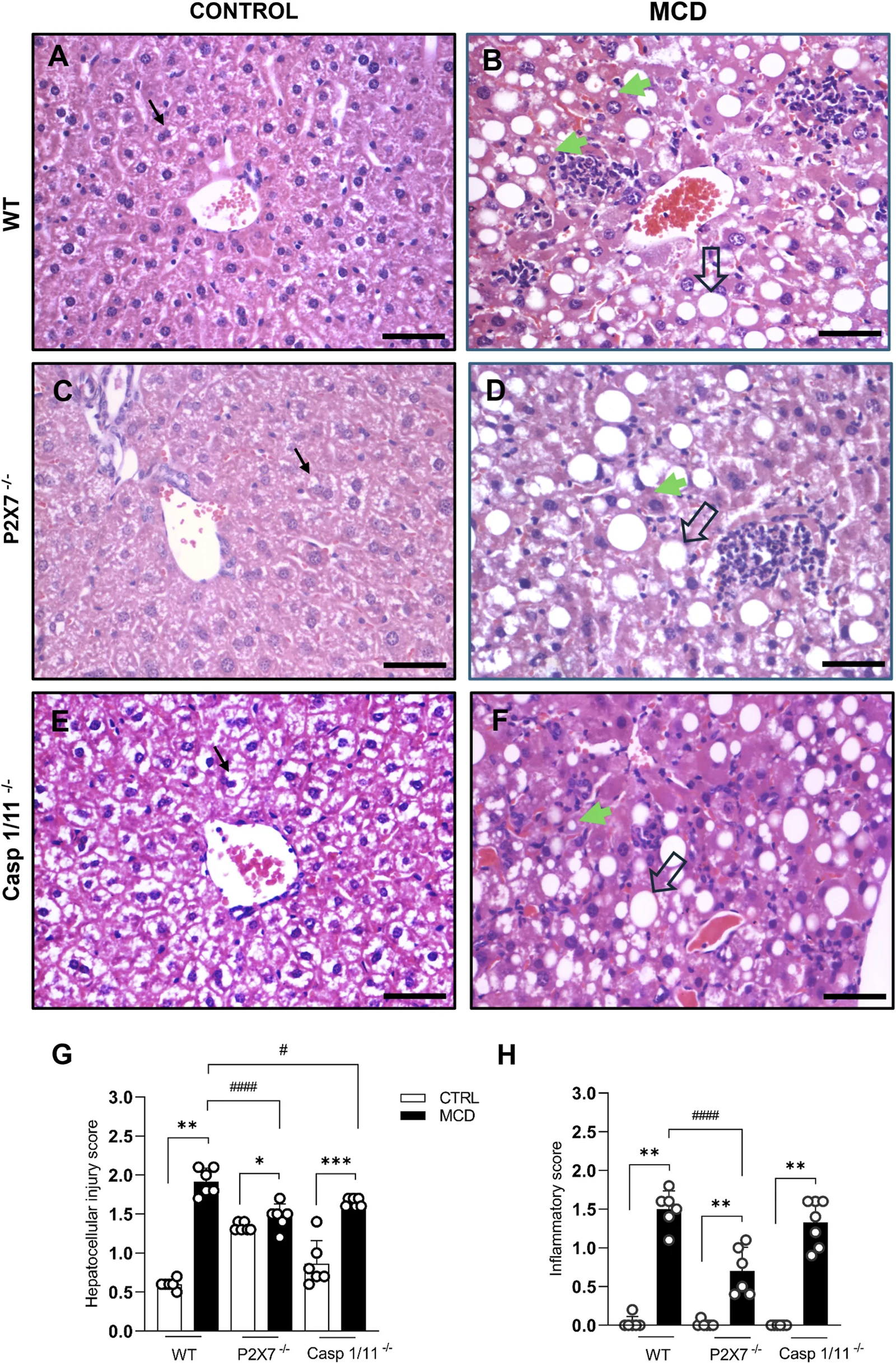
MCD diet induces hepatic histopathological alterations and inflammatory injury in WT, P2X7^−/−^, and Casp1/11^−/−^ mice. Representative histological images of liver sections from mice fed CTRL or methionine- and choline-deficient (MCD) diets for 6 weeks are shown. CTRL groups included WT CTRL **(A)** P2X7^−/−^ CTRL **(C)** and Casp1/11^−/−^ CTRL **(E)** mice, all displaying preserved hepatic architecture and normal parenchymal organization. Hepatocytes with optically clear cytoplasm were occasionally observed in CTRL animals (black arrows). MCD-fed groups included WT MCD **(B)**, P2X7^−/−^ MCD **(D)**, and Casp1/11^−/−^ MCD **(F)**, which exhibited marked hepatic steatosis characterized by macrovesicular steatosis (white arrows) and microvesicular steatosis (black arrowheads). Inflammatory cell infiltrates were observed mainly in WT and P2X7^−/−^ MCD groups (green arrowheads), whereas Casp1/11^−/−^ MCD mice exhibited reduced inflammatory infiltrates. Liver sections were stained with hematoxylin and eosin (H&E). Scale bar = 100 μm. Quantitative histopathological analysis demonstrated significantly increased hepatocellular injury scores in WT and Casp1/11^−/−^ mice following MCD diet administration, whereas no significant CTRL versus MCD difference was detected in P2X7^−/−^ mice **(G)**. Similarly, inflammatory parameter scores were significantly elevated in all MCD-fed groups compared with their respective CTRL groups **(H)**. Histopathological scoring was performed according to a modified Liang et al. (2014) classification system. Comparisons between CTRL and MCD groups within each genotype were analyzed using the Mann–Whitney test. Overall analyses of hepatocellular injury and inflammatory parameters were performed using two-way ANOVA followed with diet and genotype as factors, followed by Sidak’s multiple-comparisons test for CTRL versus MCD comparisons within each genotype. Data are presented as mean ± SEM. Statistical significance is indicated as *P < 0.05, **P < 0.01, ***P < 0.001, and ****P < 0.0001. Hashtag symbols indicate significant effects detected by two-way ANOVA. Each data point represents one animal; sample sizes ranged from 6 to 7 animals per group.

Hepatocytes from P2X7^−/−^ and Caspase-1/11^−/−^ mice fed with a control diet showed some vacuolization in the hepatocytes (Figures 2A,C,E). Moreover, WT and both knockout mice fed the MCD diet showed accumulation of lipid droplets, both microvesicular steatosis (small circular droplets) and macrovesicular steatosis (large circular droplets) (Figures 2B,D,F), and varying degrees of hepatocyte hypertrophy. The hepatocellular injury score was significantly increased following MCD diet feeding in WT and Casp1/11^−/−^ mice, whereas no significant CTRL versus MCD difference was detected in P2X7^−/−^ mice (Figure 2G). Notably, MCD-fed P2X7^−/−^ and Caspase-1/11^−/−^ mice exhibited significantly lower liver injury scores compared with wild-type animals, indicating that both P2X7 receptor and caspase-1/11 signaling contribute to hepatic damage during MASH development (Figure 2G).

WT animals fed with the MCD diet show exuberant inflammation inside liver sinusoidal zones, characterized by clusters of inflammatory cells (more than 5 myeloid cells) or by lipogranulomas/necroinflammation (Figure 2B). Some inflammatory cells can also be present in large portal spaces (data not shown). On the other hand, P2X7^−/−^ and caspase-1/11^−/−^ mice fed with the MCD diet exhibit a less conspicuous inflammatory response (Figures 2D,E). Inflammatory score demonstrated a significant increase in inflammatory cells in all groups that received the MCD diet compared with mice fed with the control diet (Figure 2H). These data indicate that MCD feeding increased hepatic inflammatory scores across genotypes, with distinct inflammatory patterns observed among the experimental groups.

Liver fibrosis is characterized by excessive extracellular matrix deposition associated with tissue remodeling and degradation, representing a common consequence of chronic liver injury, persistent inflammation, and sustained fibrogenic responses (Friedman and Pinzani, 2022; Schwabe et al., 2020). Thus, we evaluated whether the absence of either the P2X7 receptor or caspase-1/11 contributes to MCD diet-induced collagen deposition. Our results showed that the diet increased collagen fiber deposition in WT animals, whereas P2X7^−/−^ and caspase-1/11^−/−^ mice showed less collagen deposition than WT animals (Supplementary Figure S1). These data suggest that the P2X7 receptor and caspase-1/11 contribute to inflammation, in addition to participating in the fibrogenic process induced by the MCD diet.

### 3.3 P2X7 receptor regulates hepatic lipid accumulation independently of caspase-1/11 during MCD diet-induced steatosis

Steatotic livers exhibit increased triacylglycerol and cholesterol synthesis, and lipid accumulation contributes to hepatocellular injury during MASH progression (Fabbrini et al., 2008; Puri et al., 2007; Simonen et al., 2011). Consistent with these findings, our results demonstrated that MCD diet significantly increased both hepatic cholesterol and triacylglycerol levels (Figures 3A–C). These measurements were performed directly in liver tissue samples.

**FIGURE 3:**
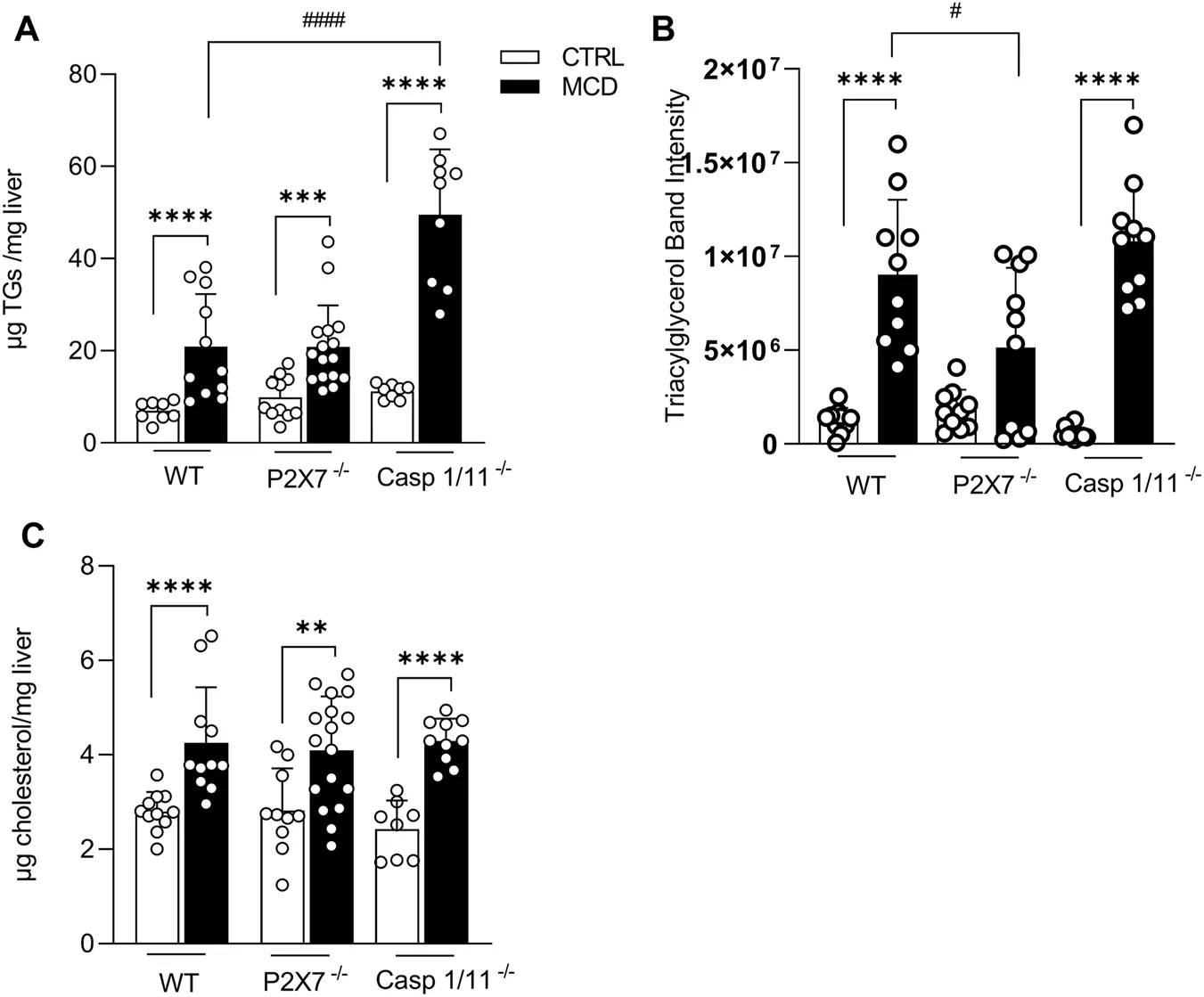
MCD diet promotes hepatic lipid accumulation and alters hepatic lipid profile in WT, P2X7^−/−^, and Casp1/11^−/−^ mice. Hepatic lipid accumulation was evaluated after 6 weeks of MCD diet treatment. Quantification of hepatic triacylglycerol content demonstrated a significant increase in triacylglycerol levels (μg TGs/mg liver) in all MCD-fed groups compared with their respective CTRL groups **(A)**. Densitometric analysis of triacylglycerol bands demonstrated increased triacylglycerol band intensity in WT and Casp1/11^−/−^ MCD-fed mice compared with their respective CTRL groups **(B)**. Hepatic cholesterol quantification revealed significantly increased cholesterol levels (μg cholesterol/mg liver) in all genotypes subjected to the MCD diet **(C)**. Experimental groups included WT CTRL, WT MCD, P2X7^−/−^ CTRL, P2X7^−/−^ MCD, Casp1/11^−/−^ CTRL, and Casp1/11^−/−^ MCD mice. Comparisons between CTRL and MCD groups within each genotype were analyzed using the Mann–Whitney test. Overall statistical analyses were performed using two-way ANOVA followed by Tukey’s multiple comparisons test. Data are presented as mean ± SEM. Statistical significance is indicated as *P < 0.05, **P < 0.01, ***P < 0.001, and ****P < 0.0001. Hashtag symbols indicate significant effects detected by two-way ANOVA. Each data point represents one animal; sample sizes ranged from 8 to 17 animals per group.

Regarding cholesterol levels (Figure 3C), all groups subjected to the MCD diet exhibited increased levels compared with animals fed a normal diet, with no marked differences among the diet-fed groups. In contrast, triacylglycerol levels (Figure 3A) were significantly elevated in all MCD diet-fed groups, with Caspase-1/11^−/−^ mice showing a markedly greater accumulation compared with the other experimental groups.

To further characterize the lipid alterations induced by the diet, we next evaluated specific lipid classes in the livers of MCD diet-fed animals. Lipid species were categorized into total monoacylglycerols (Supplementary Figure S2A), diacylglycerols (Supplementary Figure S2B), cholesteryl esters (Supplementary Figure S2C), phospholipids (Supplementary Figure S2D), and free fatty acids (Supplementary Figure S2E). Our data demonstrated that MCD diet selectively increased monoacylglycerol, diacylglycerol, and cholesteryl ester levels. Notably, the absence of the P2X7 receptor reversed the MCD diet-induced increase in monoacylglycerol and cholesteryl ester levels, whereas caspase-1/11 deficiency did not prevent the accumulation of any of these lipid species. Together, these findings indicate that the P2X7 receptor modulates lipid metabolism during MCD diet-induced steatosis independently of caspase-1/11 signaling.

### 3.4 P2X7 receptor and caspase-1/11 differentially regulate cytokine production during MCD diet-induced hepatic inflammation

Due to the accumulation of immune cells during MCD diet feeding, we hypothesized that the diet could modulate inflammatory and pro-fibrotic cytokine responses. Our results demonstrated that MCD diet increased *Il1b* mRNA expression, with a significant increase detected in WT mice. Although this increase was not significant in P2X7^−/−^ or Casp1/11^−/−^ mice, the response to MCD diet did not significantly differ among genotypes (Figure 4A).

**FIGURE 4:**
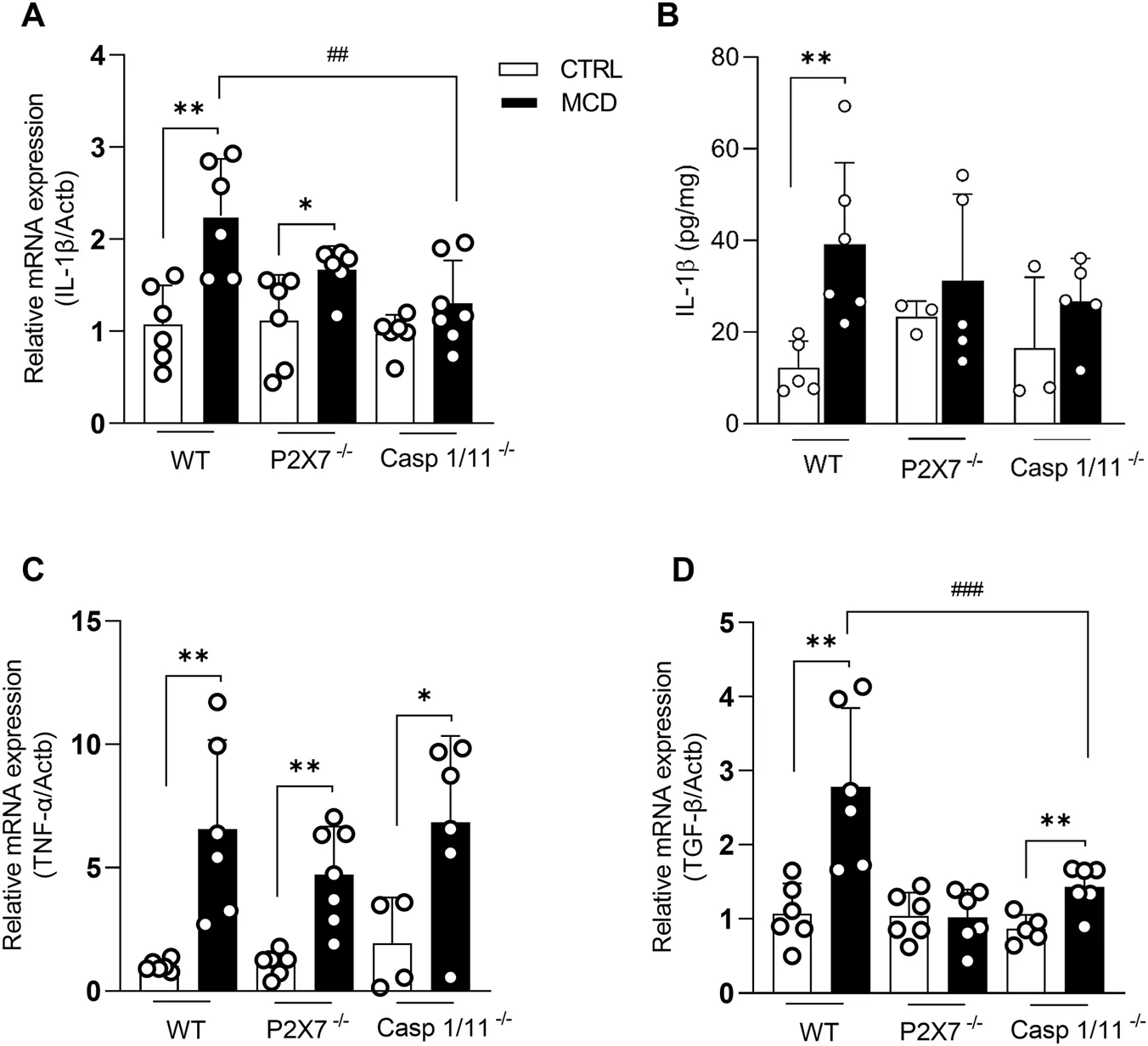
MCD diet promotes hepatic inflammatory cytokine production. Hepatic tissue samples were collected after 6 weeks of MCD diet treatment to evaluate inflammatory cytokines and *Tgfb1* expression by RT-qPCR and ELISA. Relative gene expression was normalized to *Actb* and is presented using the comparative 2^^−ΔΔCt^ method, whereas statistical analyses of RT-qPCR data were performed using the corresponding ΔCt values. *Il1b* mRNA expression was significantly increased in WT mice following MCD diet feeding, whereas no significant changes were detected in P2X7^−/−^ or Casp1/11^−/−^ mice **(A)**. IL-1β protein levels measured by ELISA were significantly elevated only in WT-MCD animals, while P2X7^−/−^ and Casp1/11^−/−^ groups showed no significant changes compared with their respective CTRL groups **(B)**. Relative *Tnf* mRNA expression was significantly increased in all genotypes following MCD diet administration **(C)**. *Tgfb1* mRNA expression was significantly increased in WT mice, whereas no significant changes were detected in P2X7^−/−^ or Casp1/11^−/−^ mice following MCD diet feeding **(D)**. Data are presented as mean ± SEM. Statistical significance between CTRL and MCD-treated groups is indicated as *P < 0.05, **P < 0.01, ***P < 0.001, and ****P < 0.0001. Hashtag symbols indicate significant effects identified by two-way ANOVA. Statistical analyses were performed using Mann–Whitney or unpaired Student’s t tests for pairwise comparisons and two-way ANOVA followed by Tukey’s multiple comparisons test. Data were obtained from 3 to 7 animals/group.

When IL-1β protein levels were evaluated, a significant increase following MCD diet feeding was detected in WT mice, whereas no significant changes were observed in P2X7^−/−^ or Casp1/11^−/−^ mice (Figure 4B). These findings suggest that P2X7 receptor signaling and caspase-1/11 may contribute to IL-1β maturation and release during MCD diet-induced hepatic inflammation, consistent with the involvement of inflammasome-related pathways.

Analysis of *Tnf* mRNA expression revealed that MCD diet increased *Tnf* expression in all experimental groups, with a comparable response among genotypes (Figure 4C).

In contrast, *Tgfb1* mRNA expression was increased in WT mice following MCD diet feeding, whereas no significant changes were observed in P2X7^−/−^ or Casp1/11^−/−^ mice. Importantly, the response to MCD diet differed significantly among genotypes, supporting genotype-dependent modulation of *Tgfb1* expression (Figure 4D).

Together, these findings indicate that the P2X7 receptor and caspase-1/11 differentially influence inflammatory and pro-fibrotic cytokine responses during MCD diet-induced liver injury, with their contribution varying according to the specific mediator evaluated.

### 3.5 MCD diet promotes purinergic remodeling and modulates inflammasome-associated pathways during hepatic inflammation

Since extracellular nucleotide signaling is closely associated with sterile inflammation and metabolic dysfunction (Vultaggio-Poma et al., 2022; Raza et al., 2025), we next investigated whether feeding an MCD diet could modulate components of the hepatic purinergic signaling pathway. Our results demonstrated that MCD diet induced a marked remodeling of the hepatic purinergic profile, with distinct patterns of regulation among individual purinergic receptors.

MCD diet feeding markedly reduced P2Y_1_ and P2Y_4_ receptor expression. P2Y_1_ downregulation was not significantly influenced by genotype, whereas the magnitude of P2Y_4_ reduction differed among genotypes (Figures 5A,B).

**FIGURE 5:**
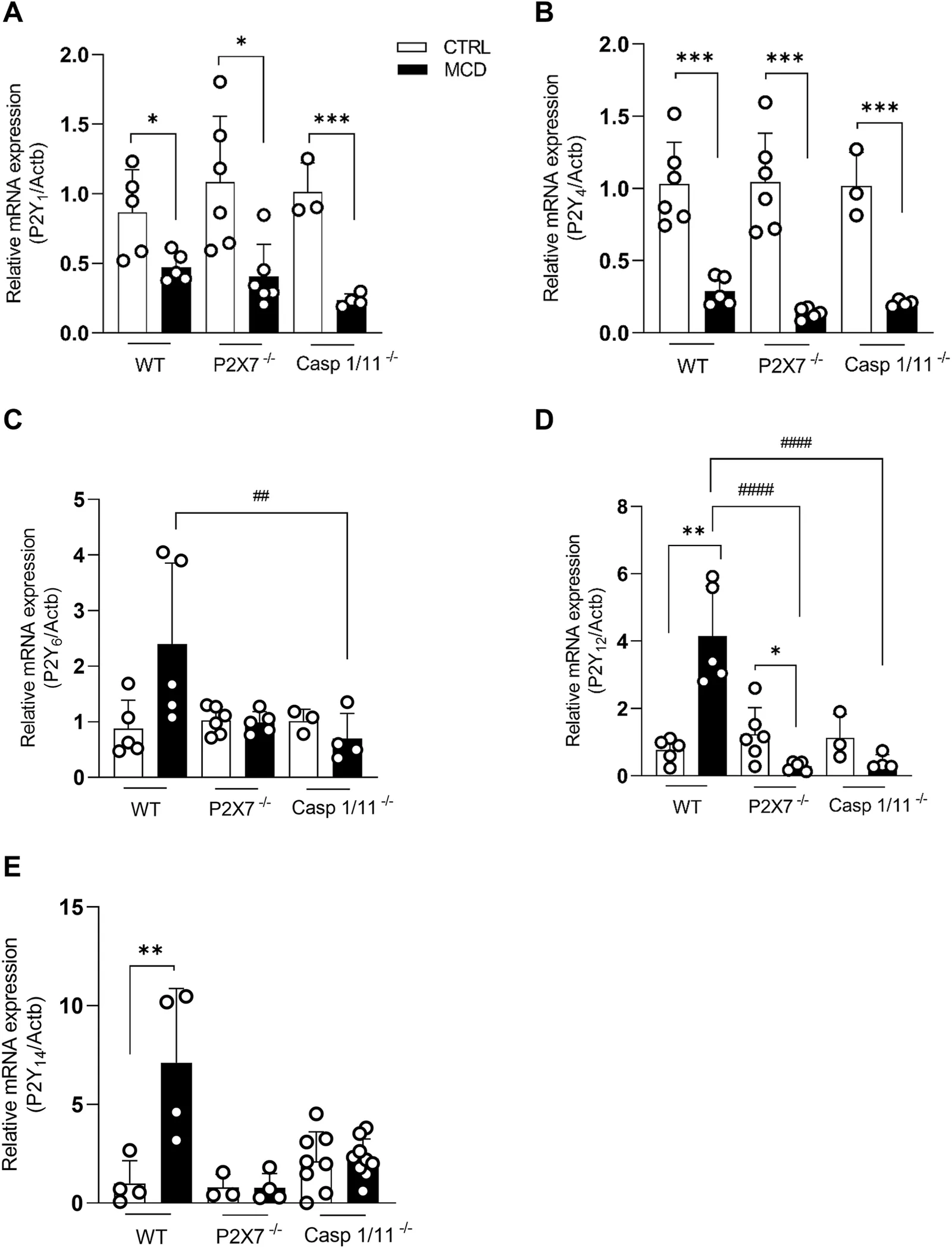
Hepatic expression of P2Y receptors in WT, P2X7^−/−^, and Casp1/11^−/−^ mice subjected to MCD diet. Relative hepatic expression of purinergic P2Y receptors was evaluated by RT-qPCR in WT, P2X7^−/−^, and Casp1/11^−/−^ mice fed CTRL or methionine- and choline-deficient (MCD) diets. **(A)** *P2ry1*, **(B)** *P2ry4*, **(C)** *P2ry6,* **(D)** *P2ry12*, and **(E)** *P2ry14*. Gene expression levels were normalized to Actb and are presented as relative expression calculated using the comparative 2^^−ΔΔCt^ method, whereas statistical analyses were performed using the corresponding ΔCt values. MCD feeding reduced *P2ry1* expression, with significant reductions detected in P2X7^−/−^ and Casp1/11^−/−^ mice **(A)**. *P2ry4* expression was significantly reduced following MCD feeding in all genotypes **(B)**. P2ry6 expression was not significantly altered by MCD feeding within any genotype **(C)**. *P2ry12* expression showed a genotype-dependent response to MCD feeding, with increased expression in WT mice and reduced expression in P2X7^−/−^ mice, whereas no significant change was detected in Casp1/11^−/−^ mice **(D)**. *P2ry14* expression was not significantly altered by MCD feeding in any genotype **(E)**. Data are presented as mean ± SEM. Statistical analyses were performed using two-way ANOVA with diet and genotype as factors, followed by Tukey’s multiple comparisons test. Statistical significance is indicated as P < 0.05, P < 0.01, P < 0.001, and P < 0.0001; ns, not significant. Data were obtained from 3 to 7 animals/group.

In contrast, P2Y_6_ expression differed among genotypes but was not significantly altered by MCD feeding within the individual groups (Figure 5C). P2Y_12_ showed a pronounced genotype-dependent response to MCD feeding, with increased expression in WT mice and an opposite response in P2X7^−/−^ mice, whereas no significant change was detected in Casp1/11^−/−^ mice (Figure 5D). P2Y_14_ expression was not significantly altered by MCD feeding in any of the experimental groups (Figure 5E). Given that P2Y_6_, P2Y_12_, and P2Y_14_ have been implicated in inflammatory cell activation, leukocyte recruitment, and innate immune responses (Haynes et al., 2006; Uratsuji et al., 2012; da Silva Ebone et al., 2026), these findings indicate that genotype-dependent differences were observed in the transcript levels of specific purinergic components, rather than a uniform alteration of the hepatic purinergic profile across genotypes.

Finally, we analyzed the expression of the ectonucleotidases CD39 (*Entpd1*) and CD73 (*Nt5e*), which are responsible for extracellular ATP degradation and adenosine generation. MCD feeding significantly increased *Entpd1* expression in WT mice, whereas no significant changes were observed in P2X7^−/−^ or Casp1/11^*−/−*^ mice. Similarly, *Nt5e* expression responded differently to MCD feeding according to genotype, increasing in WT mice but decreasing in P2X7^−/−^ mice, while remaining unchanged in Casp1/11^*−/−*^ mice (Supplementary Figure S3A,B). These results indicate that MCD-induced liver injury is associated with genotype-dependent alterations in the expression of ectonucleotidases involved in extracellular ATP metabolism, rather than a uniform upregulation of these compensatory pathways. The differential regulation of *Entpd1* and *Nt5e* among genotypes suggests that P2X7 receptor and caspase-1/11 signaling may influence the hepatic response to extracellular nucleotide metabolism during MCD-induced injury.

Together, these findings demonstrate that MCD diet-induced steatohepatitis is associated with substantial remodeling of the hepatic purinergic network. Importantly, the contribution of P2X7 receptor and caspase-1/11 signaling varied among individual purinergic components, with particularly pronounced genotype-dependent responses observed for P2Y_12_, CD39, and CD73. These findings suggest that P2X7 receptor and caspase-1/11 contribute selectively to the remodeling of hepatic purinergic signaling during MCD-induced liver injury.

## 4 Discussion

The MCD diet is a well-established experimental model for MASH in mice, as it rapidly induces hepatic steatosis, liver injury, inflammation, and fibrosis, even in the absence of insulin resistance or hyperglycemia (Hebbard and George, 2011; Rinella and Green, 2004; Santhekadur et al., 2018). This diet contains approximately 40% sucrose and 10% fat but lacks methionine and choline, two essential factors for hepatic mitochondrial β-oxidation and very low-density lipoprotein synthesis (Anstee and Goldin, 2006). Its metabolic impact includes reduced glycogen stores, protein peroxidation (Freitas et al., 2016), increased ROS production, mitochondrial DNA damage, and apoptosis (Gao et al., 2004). Similarly, choline-deficient diets in humans have also been associated with the development of fatty liver disease (Kohlmeier et al., 2005). Together, these findings emphasize the critical role of methionine and choline deficiency in driving hepatic metabolic dysfunction and steatosis in both mice and humans.

Beyond these metabolic alterations, inflammatory mechanisms have also been implicated in liver pathology. Previous studies have reported increased expression of NLRP3 inflammasome components in the context of liver disease (Csak et al., 2014; Szabo and Petrasek, 2015). Diets enriched in fat and carbohydrates have been shown to elevate hepatic levels of triacylglycerol, cholesterol (Tall and Yvan-Charvet, 2015), and succinate (Jakobsdottir et al., 2013), with succinate functioning as a signal for inflammasome activation (Tannahill et al., 2013). In this context, the NLRP3 inflammasome can also be triggered by cellular stress and damage-associated molecular patterns (DAMPs), ROS, and viral or chemical stimuli, leading to IL-1β and IL-18 maturation and pyroptosis, processes that play critical roles in both acute and chronic liver injury (Sayaf et al., 2024). Importantly, IL-1β has been directly associated with MASH progression (Mridha et al., 2017; Schroder and Tschopp, 2010).

In agreement with these observations, our results confirmed that MCD diet induces significant weight loss in mice (Friedman et al., 2018). Notably, this effect occurred independently of both P2X7 receptor activation and caspase-1/11 signaling. In addition, MCD diet feeding promoted a characteristic yellowish discoloration of the liver accompanied by reduced liver weight, effects that were also unaffected by P2X7 receptor or caspase-1/11 deficiency. These findings suggest that the major macroscopic alterations induced by MCD diet are predominantly driven by metabolic disturbances rather than by P2X7 receptor- and inflammasome-associated signaling.

Fatty liver disease initially develops as simple steatosis, characterized by excessive lipid accumulation in hepatocytes. As the disease progresses, hepatocytes undergo cell death due to lipid accumulation, leading to immune cell recruitment and inflammation. Hepatocyte injury contributes to the development of MASH, while persistent liver damage promotes hepatic stellate cell activation, collagen deposition, and fibrosis. Histologically, this process is characterized by steatosis, inflammatory infiltrates, hepatocyte ballooning, and fibrosis (Peverill et al., 2014). Consistent with this pathological profile, our histological analyses demonstrated that feeding an MCD diet induced marked hepatic steatosis accompanied by inflammatory infiltrates and hepatocellular injury.

P2X7 receptor functions as a danger sensor that detects extracellular ATP released during tissue injury and inflammation, promoting NLRP3 inflammasome assembly, caspase-1 activation, and IL-1β maturation (Sanz and Di Virgilio, 2000; Mariathasan et al., 2004; Mariathasan and Monack, 2007; Rubartelli and Lotze, 2007). Inflammatory priming by PAMPs, such as LPS, induces pro-IL-1β synthesis, whereas ATP acts as a DAMP signal that triggers its release through P2X7 receptor activation (Ferrari et al., 1997; Mariathasan et al., 2004; Rubartelli and Lotze, 2007). IL-1β can subsequently enhance TGF-β1 signaling, a key mediator of fibrogenesis (LeRoy et al., 1990). Fibrosis is characterized by excessive extracellular matrix deposition resulting from dysregulated wound-healing responses (Eckes et al., 2000; Wynn, 2008). Since IL-1β promotes collagen expression and can amplify its own production, sustained inflammasome activation may contribute to persistent TGF-β1 activity and fibrotic progression (Postlethwaite et al., 1988). Therefore, P2X7 receptor may represent an important regulator of profibrotic pathways and a potential therapeutic target in fibrotic diseases. Based on that, our data demonstrated that the absence of either the P2X7 receptor or caspase-1/11 led to a reduction in collagen deposition, a hallmark feature of MASH (Schwabe et al., 2020; Friedman and Pinzani, 2022).

The differential TGF-β response observed among genotypes suggests that P2X7 receptor signaling and caspase-1/11 may contribute to the regulation of TGF-β during MCD-induced liver injury. While MCD diet increased TGF-β expression in WT mice, this response was not observed in P2X7^−/−^ or Casp1/11^−/−^ mice. One possible mechanism may involve activation of the NLRP3 inflammasome, as P2X7 receptor is a well-established upstream signal for NLRP3 activation, leading to caspase-1 activation and the release of pro-inflammatory mediators that may contribute to pro-fibrotic responses. The absence of a significant MCD-induced increase in TGF-β expression in Casp1/11^−/−^ mice is consistent with the possibility that inflammasome-dependent signaling contributes to TGF-β regulation.

However, P2X7 receptor signaling may also influence TGF-β expression through mechanisms that extend beyond caspase-1/11-dependent inflammasome signaling. In addition to its role in NLRP3 inflammasome activation, P2X7 receptor has been implicated in ATP-dependent signaling pathways associated with hepatic stellate cell activation and fibrogenic responses, including PKC/GSK3β signaling (Wu et al., 2017). Although Wu et al. (2017) did not directly demonstrate that PKC/GSK3β activation induces TGF-β production, activation of this pathway increased markers of hepatic stellate cell activation, including α-SMA and collagen I. Activated hepatic stellate cells are also capable of producing and secreting TGF-β1 and play a central role in hepatic fibrogenesis (Arias et al., 2002; Dooley et al., 2001). Thus, these findings raise the possibility that P2X7 receptor-dependent signaling may indirectly influence TGF-β expression through effects on hepatic stellate cell activation, potentially involving mechanisms beyond caspase-1/11 signaling.

Nevertheless, our findings do not establish whether the influence of P2X7 receptor signaling on TGF-β expression is mediated predominantly through the NLRP3 inflammasome or through parallel inflammasome-independent pathways. Further studies directly assessing NLRP3 activation, caspase-1/11 signaling, and downstream P2X7 receptor-dependent pathways will be necessary to define the mechanisms underlying TGF-β regulation in this model and to determine the relative contribution of inflammasome-dependent and -independent mechanisms.

These alterations were accompanied by significant accumulation of triacylglycerol and cholesterol in liver tissue, further supporting the establishment of a steatotic and inflammatory hepatic microenvironment. Interestingly, caspase-1/11^−/−^ mice exhibited markedly higher triacylglycerol accumulation compared with WT and P2X7^−/−^ animals, suggesting that inflammasome-associated signaling may differentially influence hepatic lipid handling during MCD diet-induced liver injury. Together, these findings reinforce the close relationship between metabolic dysregulation, lipid accumulation, and inflammatory responses during steatohepatitis progression.

In parallel, our results demonstrated that MCD diet feeding induced a marked inflammatory response accompanied by alterations in IL-1β expression and production. IL-1β mRNA expression was increased in WT mice following MCD diet feeding, whereas no significant changes were detected in P2X7^−/−^ or Casp1/11^−/−^ mice. However, the overall response to MCD diet was not significantly different among genotypes. At the protein level, only WT animals exhibited increased IL-1β levels following MCD diet feeding, while no significant increase was observed in P2X7^−/−^ or Casp1/11^−/−^ mice. This pattern is consistent with a potential contribution of P2X7 receptor signaling and caspase-1/11 to IL-1β maturation and release during MCD diet-induced liver injury. These findings suggest that, while metabolic alterations induced by MCD diet occur independently of inflammasome activation, inflammasome-associated signaling may contribute to the inflammatory response accompanying steatohepatitis progression. Since extracellular nucleotide signaling is closely associated with sterile inflammation and metabolic dysfunction (Raza et al., 2025; Vultaggio-Poma et al., 2022), we next investigated whether MCD diet feeding could modulate components of the hepatic purinergic signaling pathway. Our results demonstrated that MCD diet promoted remodeling of the hepatic purinergic profile, with distinct patterns of regulation among individual P2Y receptors. P2Y_1_ and P2Y_4_ expression were reduced following MCD diet feeding, although the response of P2Y_4_ varied among genotypes. In contrast, P2Y_6_ expression differed among genotypes but was not significantly altered by MCD diet feeding, while P2Y_12_ displayed a pronounced genotype-dependent response. P2Y_14_ expression remained largely unchanged following MCD diet feeding. Together, these findings suggest that P2X7 receptor and caspase-1/11 signaling selectively influence specific components of the hepatic purinergic network rather than uniformly regulating the purinergic response during MCD-induced liver injury. However, because these analyses were performed in whole-liver homogenates, the observed transcript changes may reflect both altered gene expression and changes in cellular composition resulting from inflammatory cell infiltration.

These findings are supported by previous studies implicating purinergic receptors in hepatic inflammation and fibrosis. Although increased P2Y_6_ receptor expression has been described in experimental MASH models, this receptor does not appear to directly contribute to hepatic injury progression (Nishiyama et al., 2023). In contrast, P2Y_12_ receptor signaling was shown to exacerbate autoimmune hepatitis through hexokinase 2-dependent glycolysis in T cells (Zhuang et al., 2023), while P2Y_14_ receptor activation was associated with the coupling of hepatocyte injury to hepatic stellate cell activation and liver fibrogenesis (Mederacke et al., 2022). Together, these observations support the possibility that the distinct changes in purinergic receptor expression observed in our model may participate in the inflammatory and fibrogenic environment associated with MCD-induced liver injury.

Importantly, although the control diet used in the present study was not ingredient-matched to the MCD formulation, the systemic and hepatic responses observed were comparable to those reported in previous studies using nutrient-matched control diets. Studies comparing the MCD formulation with its methionine- and choline-sufficient matched control have reported similar alterations in body weight, liver injury, and hepatic inflammation, including steatosis and inflammatory foci (Beaven et al., 2011; Kirsch et al., 2003; Yang et al., 2019). Thus, despite differences in the overall composition of the control diet, the main phenotypic changes observed in our study are in agreement with those reported using an ingredient-matched control. Importantly, these findings should be interpreted as reflecting the biological responses associated with the two dietary formulations evaluated and should not be taken as evidence that methionine and choline deficiency alone accounts for the observed phenotypes. Moreover, sex is an important biological determinant of metabolic and hepatic responses to dietary interventions, and previous studies using the MCD model have demonstrated sex-dependent differences in the severity of hepatic injury and inflammatory responses. In particular, male C57BL/6 mice have been reported to develop more pronounced hepatic inflammation, necrosis, lipid peroxidation, and ultrastructural injury in response to MCD feeding (Kirsch et al., 2003). Similarly, sex-specific differences in hepatic and metabolic responses to MCD feeding have been reported in subsequent studies (Lee et al., 2016). Based on this evidence, male mice were selected in the present study to provide a consistent and robust hepatic phenotype for investigating the contribution of P2X7 receptor and caspase-1/11 signaling to MCD-associated steatohepatitis.

Collectively, our findings identify P2X7 receptor signaling as an important modulator of hepatic inflammation and collagen deposition in the dietary model evaluated in this study. P2X7^−/−^ and Casp1/11^−/−^ mice exhibited distinct patterns of liver injury and inflammatory responses, revealing differential contributions of P2X7 receptor signaling and caspase-1/11 to the hepatic response to the dietary intervention. Importantly, our study extends current knowledge by demonstrating that these responses are accompanied by broader remodeling of the hepatic purinergic network, including differential regulation of P2Y receptor transcripts. Together, these findings establish P2X7 receptor-associated signaling as an important component of the hepatic inflammatory response and reveal that its effects are integrated with broader alterations in the purinergic environment during chronic liver inflammation, highlighting P2Y receptors as additional pathways of interest in MASH.

The protective phenotype observed in P2X7^−/−^ mice further supports the involvement of P2X7 receptor signaling in hepatic injury and inflammation, while the distinct responses observed in Casp1/11^−/−^ mice underscore the differential contribution of inflammasome-associated signaling to these processes. However, the present findings do not establish the therapeutic efficacy of pharmacological P2X7 receptor inhibition. Further studies using selective P2X7 receptor antagonists will be important to define the specific mechanisms involved and determine the translational potential of targeting P2X7 receptor and related purinergic pathways in MASH.

## Data Availability

The raw data supporting the conclusions of this article will be made available by the authors, without undue reservation.
