# Supplementary material for: P2X7 receptor contributes to hepatic inflammation and collagen deposition in methionine- and choline-deficient diet-fed mice
Source: Front Pharmacol. 2026 Oct 7;17:1909005. doi: 10.3389/fphar.2026.1909005 (PMC13683839; doi:10.3389/fphar.2026.1909005)
Supplement: Supplementary file 1 [file Supplementaryfile1.docx]

Supplementary Material

**1 Figures**

**Supplementary Figure 1. P2X7 receptor and caspase-1/11 deficiency attenuate MCD diet-induced liver collagen deposition.** Representative histological images of liver sections from mice fed CTRL or methionine- and choline-deficient (MCD) diets for 6 weeks are shown. CTRL groups included WT CTRL **(A),** P2X7^−/−^ CTRL **(C),** and Casp1/11^−/−^ CTRL **(E)** mice displaying preserved hepatic architecture with mild basal collagen fibers in the liver parenchyma. MCD-fed groups included WT MCD **(B),** P2X7^−/−^ MCD **(D),** and Casp1/11^−/−^ MCD **(F).** WT MCD exhibited increased collagen deposition associated with inflammatory cells around hepatic steatosis as well as in Casp1/11^−/−^ MCD animal. Collagen fibers are indicated by black arrows. Quantification of Picrosirius Red-positive area. Sixteen fields were analyzed per animal, and each point represents the mean of the analyzed fields (n = 5–6 animals/group). Data are presented as mean ± SEM. Statistical analysis was performed using two-way ANOVA followed by Tukey’s multiple-comparisons test. ****P < 0.0001. Liver sections were stained with Picrosirius Red. Scale bar = 100 μm.

.

**Supplementary Figure 2. Quantification and statistical analysis of hepatic lipid band intensities in WT,** $\text{P2X7}^{\mathbf{-/-}}$**, and** $\text{Casp1/11}^{\mathbf{-/-}}$ **mice subjected to MCD die**t. **(A)** Monoacylglycerol band intensity; **(B)** cholesterol/diacylglycerol band intensity; **(C)** cholesteryl ester band intensity; **(D)** total phospholipid band intensity; and **(E)** free fatty acid band intensity in liver samples from WT, $\text{P2X7}^{-/-}$, and $\text{Casp1/11}^{-/-}$ mice fed CTRL or methionine- and choline-deficient (MCD) diets. Lipid fractions were quantified by densitometric analysis and expressed as arbitrary units of band intensity. Monoacylglycerol and cholesteryl ester band intensities were significantly increased in WT and $\text{Casp1/11}^{-/-}$ MCD-fed mice, whereas cholesterol/diacylglycerol band intensity was elevated in all genotypes following MCD diet administration. No significant differences were observed for total phospholipid or free fatty acid band intensities. Comparisons between CTRL and MCD groups within each genotype were performed using unpaired Student's t test or Mann–Whitney test according to data distribution. For cholesterol/diacylglycerol band intensity, two-way ANOVA followed by Tukey's multiple comparisons test was additionally performed to evaluate the effects of diet and genotype. Data are presented as mean $\pm$ SEM for parametric analyses or median values for nonparametric analyses. Statistical significance was considered at $\text{P}<0.05$. $\text{ns}$, not significant; $*\text{P}<0.05$, $\text{P}<0.01$, $***\text{P}<0.001$, and $****\text{P}<0.0001$. Sample sizes varied between groups ($\text{n}=8\text{-}12\text{ animals/group}$), with individual data points representing individual animals.

.

**Supplementary Figure 3. Hepatic expression of ectonucleotidases CD39 and CD73 in WT,** $\text{P2X7}^{\boldsymbol{-/-}}$**, and** $\text{Casp1/11}^{\boldsymbol{-/-}}$ **mice subjected to MCD-induced NASH**. **(A)** Relative hepatic *Entpd1* (CD39) and **(B)** *Nt5e* (CD73) mRNA expression were evaluated by RT-qPCR in WT, $\text{P2X7}^{-/-}$, and $\text{Casp1/11}^{-/-}$ mice fed CTRL or methionine- and choline-deficient (MCD) diets. Gene expression levels were normalized to *Actb* and are presented as relative expression calculated using the comparative $2^{-\Delta\Delta\text{Ct}}$ method, whereas statistical analyses were performed using the corresponding $\Delta\text{Ct}$ values. MCD feeding significantly increased *Entpd1* expression in WT mice, whereas no significant changes were observed in $\text{P2X7}^{-/-}$ or $\text{Casp1/11}^{-/-}$ mice **(A)**. For *Nt5e*, MCD feeding significantly increased expression in WT mice and reduced expression in $\text{P2X7}^{-/-}$ mice, whereas no significant change was observed in $\text{Casp1/11}^{-/-}$ mice **(B)**. Data are presented as mean $\pm$ SEM. Statistical analyses were performed using two-way ANOVA with diet and genotype as factors, followed by Tukey's multiple comparisons test. Statistical significance was considered at $P<0.05$. Significance levels are represented as $P<0.05$, $P<0.01$, $P<0.001$, and $\text{P}<0.0001$; ns, not significant. Sample sizes varied between groups ($\text{n}=4\text{-}9\text{ animals/group}$), with individual data points representing individual animals.
